# ZBP1 and TRIF trigger lethal necroptosis in mice lacking caspase-8 and TNFR1

**DOI:** 10.1038/s41418-024-01286-6

**Published:** 2024-03-28

**Authors:** Margaret Solon, Nianfeng Ge, Shannon Hambro, Susan Haller, Jian Jiang, Miriam Baca, Jessica Preston, Allie Maltzman, Katherine E. Wickliffe, Yuxin Liang, Rohit Reja, Dorothee Nickles, Kim Newton, Joshua D. Webster

**Affiliations:** 1 Department of Pathology, Genentech, 1 DNA Way, South San Francisco, CA 94080 USA; 2 Department of Physiological Chemistry, Genentech, 1 DNA Way, South San Francisco, CA 94080 USA; 3 Department of Microchemistry, Proteomics, Lipidomics, and Next Generation Sequencing, Genentech, 1 DNA Way, South San Francisco, CA 94080 USA; 4 Department of Oncology Bioinformatics, Genentech, 1 DNA Way, South San Francisco, CA 94080 USA; 5 Department of Translational Oncology, Genentech, 1 DNA Way, South San Francisco, CA 94080 USA

**Keywords:** Cell death and immune response, Immune cell death

## Abstract

Necroptosis is a lytic form of cell death that is mediated by the kinase RIPK3 and the pseudokinase MLKL when caspase-8 is inhibited downstream of death receptors, toll-like receptor 3 (TLR3), TLR4, and the intracellular Z-form nucleic acid sensor ZBP1. Oligomerization and activation of RIPK3 is driven by interactions with the kinase RIPK1, the TLR adaptor TRIF, or ZBP1. In this study, we use immunohistochemistry (IHC) and in situ hybridization (ISH) assays to generate a tissue atlas characterizing RIPK1, RIPK3, *Mlkl*, and ZBP1 expression in mouse tissues. RIPK1, RIPK3, and *Mlkl* were co-expressed in most immune cell populations, endothelial cells, and many barrier epithelia. ZBP1 was expressed in many immune populations, but had more variable expression in epithelia compared to RIPK1, RIPK3, and *Mlkl*. Intriguingly, expression of ZBP1 was elevated in *Casp8*^*−/−*^ *Tnfr1*^*−/−*^ embryos prior to their succumbing to aberrant necroptosis around embryonic day 15 (E15). ZBP1 contributed to this embryonic lethality because rare *Casp8*^*−/−*^ *Tnfr1*^*−/−*^ *Zbp1*^*−/−*^ mice survived until after birth. Necroptosis mediated by TRIF contributed to the demise of *Casp8*^*−/−*^ *Tnfr1*^*−/−*^ *Zbp1*^*−/−*^ pups in the perinatal period. Of note, *Casp8*^*−/−*^ *Tnfr1*^*−/−*^ *Trif*^*−/−*^ *Zbp1*^*−/−*^ mice exhibited autoinflammation and morbidity, typically within 5–7 weeks of being born, which is not seen in *Casp8*^*−/−*^ *Ripk1*^*−/−*^ *Trif*^*−/−*^ *Zbp1*^*−/−*^, *Casp8*^*−/−*^ *Ripk3*^*−/−*^, or *Casp8*^*−/−*^ *Mlkl*^*−/−*^ mice. Therefore, after birth, loss of caspase-8 probably unleashes RIPK1-dependent necroptosis driven by death receptors other than TNFR1.

## Introduction

Inactivation of caspase-8 promotes necroptosis, a lytic cell death induced by death ligands such as tumor necrosis factor (TNF) [1–6]. Caspase-8 suppresses necroptosis by cleaving RIPK1 within death receptor signaling complexes [7–10]. RIPK1 promotes necroptosis by recruiting RIPK3 [11, 12]. Oligomerized RIPK3 phosphorylates MLKL, prompting MLKL translocation to the plasma membrane and cell lysis [13]. Caspase-8 also suppresses activation of RIPK3 by TRIF and ZBP1 [14]. TLR3 and TLR4 engage TRIF to activate RIPK3 [15, 16], whereas ZBP1 activates RIPK3 in response to Z-form nucleic acids [17]. TRIF and ZBP1 can activate RIPK3 in the absence of RIPK1 [14, 16], so it is unclear if caspase-8 cleavage of RIPK1 suppresses necroptosis triggered by TRIF or ZBP1.

Caspase-8 is essential for mouse development. Mice lacking caspase-8 or expressing inactive caspase-8 die of RIPK1-, RIPK3-, and MLKL-dependent necroptosis around E11 [5, 6, 8, 18–23]. Endothelial cells lacking caspase-8 are particularly susceptible to necroptosis [24]. The trigger of necroptosis in *Casp8*^*−/−*^ embryos appears to be TNF receptor 1 (TNFR1) because *Casp8*^*−/−*^ *Tnfr1*^*−/−*^ embryos survive beyond E11, although they do not survive to birth [19]. *Casp8*^*−/−*^ *Zbp1*^*−/−*^ and *Casp8*^*−/−*^ *Trif*^*−/−*^ embryos are reported to be indistinguishable from *Casp8*^*−/−*^ embryos [16, 25]. When deletion of *Casp8* is restricted to certain cell types, mice can develop necroptosis-driven lesions after birth [26–28]. To gain further insights into the biology of RIPK1, ZBP1, RIPK3, and MLKL, we generated a tissue atlas to compare their expression patterns in mouse tissues using IHC and ISH assays. In the course of these studies, we found that expression of ZBP1 was more robust in E12.5 *Casp8*^*−/−*^ *Tnfr1*^*−/−*^ embryos than in control littermates. We show that *Casp8*^*−/−*^ *Tnfr1*^*−/−*^ *Zbp1*^*−/−*^ mice survive to birth similar to *Casp8*^*−/−*^ *Ripk1*^*−/−*^ mice [19–21]. Co-deletion of *Trif* and *Zbp1* allowed *Casp8*^*−/−*^ *Tnfr1*^*−/−*^ *Zbp1*^*−/−*^ *Trif*^*−/−*^ mice to survive past weaning. Thus, caspase-8 is a suppressor of ZBP1-driven necroptosis in the sterile environment of the embryo, in addition to being a suppressor of ZBP1-driven necroptosis in adult intestinal epithelium [26].

## Results

### IHC and ISH validation

**Fig. 1:**
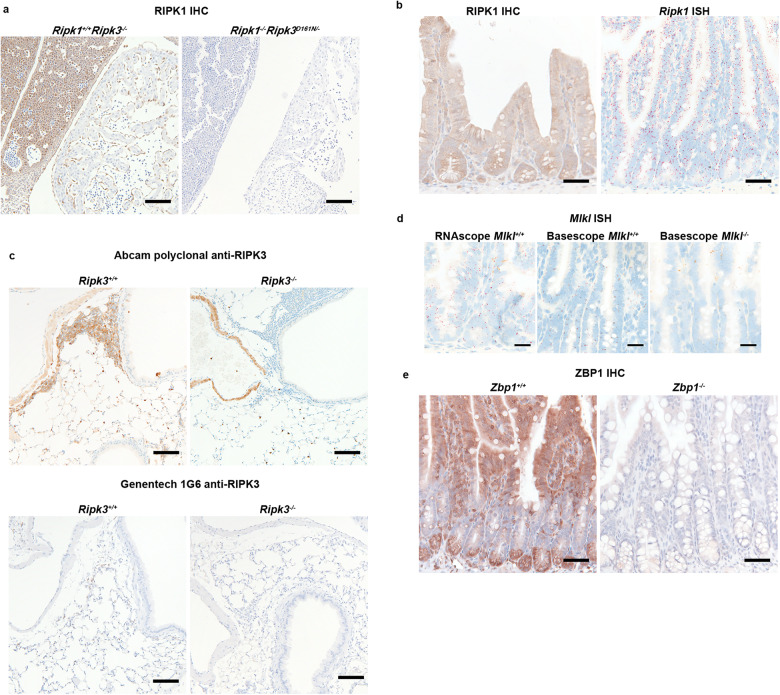
Assays for assessing RIPK1, *Ripk1*, RIPK3, *Mlkl*, and ZBP1 expression in mouse tissues. **a** RIPK1 IHC in E11.5 *Ripk1*^*+/+*^*Ripk3*^*−/−*^ (*n* = 1) and *Ripk1*^*−/−*^*Ripk3*^*D161N/−*^ (*n* = 1) embryos. Scale bar, 100 μm. **b** RIPK1 IHC (left column) and *Ripk1* ISH (right column) in WT small intestines. Scale bar, 50 µm. Results representative of 5 mice. **c** RIPK3 IHC in lung. Note the non-specific labeling of *Ripk3*^*−/−*^ striated muscle and granulocytes with the Abcam polyclonal antibody, which is not seen with the Genentech 1G6 antibody. Scale bar, 100 µm. Results representative of 5 WT and 3 *Ripk3*^*−/−*^ mice. **d** *Mlkl* ISH in small intestine with RNAscope probes targeting the full transcript (left) and a Basescope probe (center and right) that targets exon 3, which is deleted in *Mlkl*^*−/−*^ mice. Scale bar, 25 µm. Results representative of 5 WT and 3 *Mlkl*^*−/−*^ mice. **e** ZBP1 IHC in small intestine. Scale bar, 50 µm. Results representative of 5 WT and 4 *Zbp1*^*−/−*^ mice.

We assessed RIPK1, *Ripk1*, RIPK3, *Mlkl*, and ZBP1 expression in wild-type (WT) mouse tissues by IHC or ISH, using tissues from knockout mice as negative controls. The specificity of RIPK1 immunolabeling with 10C7 rat anti-mouse RIPK1 antibody was confirmed in E11.5 *Ripk1*^*+/+*^*Ripk3*^*−/−*^ and *Ripk1*^*−/−*^*Ripk3*^*D161N/-*^ embryos [29]. Endothelial cells and the liver were labeled in *Ripk1*^*+/+*^, but not *Ripk1*^*−/−*^ embryos (Fig. 1a). *Ripk1*^*−/−*^ mice die perinatally [30], so we validated our RIPK1 IHC in a subset of adult tissues by *Ripk1* ISH (Fig. 1b and Supplementary Fig. S1).

Two anti-RIPK3 antibodies had unique off-target labeling patterns in *Ripk3*^*−/−*^ tissues, so we used them to cross-validate specific RIPK3 labeling in WT tissues. Rat 1G6 anti-mouse RIPK3 antibody [29] differentially labeled WT and *Ripk3*^*−/−*^ tissues but exhibited weak labeling in *Ripk3*^*−/−*^ intestinal epithelium and renal tubules (Supplementary Fig. S2a). A rabbit anti-RIPK3 antibody (Abcam ab62344) was more sensitive than 1G6 for RIPK3 detection, but it also labeled *Ripk3*^*−/−*^ striated muscles (skeletal and cardiac), cilia, granulocytes, stratum corneum of squamous epithelium, tracheal submucosal glands, and endometrial glands (Fig. 1c and Supplementary Fig. S2b).

We evaluated three anti-MLKL antibodies by IHC (Cell Signaling Technology #28640, Lifespan Biosciences LS-C334151, and Genentech 1G12), but saw no differential labeling between WT and *Mlkl*^*−/−*^ tissues. An RNAscope ISH probe targeting full-length *Mlkl* also labeled *Mlkl*^*−/−*^ tissues lacking exon 3 [31] (Supplementary Fig. S3a). However, a Basescope probe composed of a single z-z pair targeting the deleted exon validated the RNAscope labeling (Fig. 1d and Supplementary Fig. S3b). ZBP1 IHC with GN58.3 rat anti-mouse ZBP1 antibody gave weak background signal in some *Zbp1*^*−/−*^ tissues, but strong differential labeling above background in WT tissues (Fig. 1e and Supplementary Fig. S4). GN58.3 also detected ZBP1 by western blotting (Supplementary Fig. S5).

### Tissue expression patterns of RIPK1, RIPK3, and *Mlkl*

**Fig. 2:**
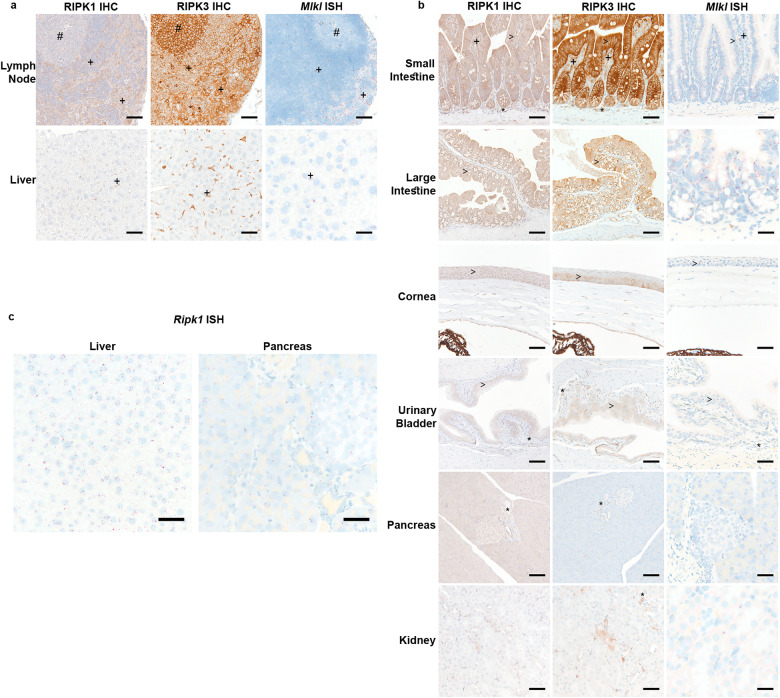
Expression of RIPK1, RIPK3, and *Mlkl* in mouse tissues. **a**, **b** RIPK1 IHC, RIPK3 IHC, and *Mlkl* ISH in WT mouse tissues. Scales bars, 25 µm (*Mlkl*: large intestine), 50 µm (RIPK1, RIPK3, and *Mlkl*: lymph node, liver, small intestine, and eye; *Mlkl*: urinary bladder and pancreas), or 100 µm (RIPK1 and RIPK3: large intestine and urinary bladder). Results representative of 5 mice. #, denotes germinal centers in the lymph nodes; >, denotes epithelial labeling; *, denotes endothelial labeling; and +, highlights immune cell labeling. **c** *Ripk1* ISH in WT mouse tissues. Scale bars, 50 µm. Results representative of 5 mice.

RIPK1, RIPK3, and *Mlkl* were most consistently expressed in adult endothelium, immune cells, and barrier epithelia (Fig. 2a, b, Supplementary Figs. S6 and S7; Supplementary Table S1). RIPK1 and *Mlkl* were detected in myeloid-rich compartments (splenic red pulp and lymph node medulla) and T-cell rich areas (lymph node paracortex and splenic periarteriolar lymphoid sheaths). They exhibited weaker labeling in lymphoid follicles, particularly in germinal centers. By contrast, RIPK3 was prominent in myeloid regions and germinal centers (Fig. 2a and Supplementary Fig. S6). In many tissues, interstitial cells expressed RIPK1 and RIPK3, including Kupffer cells and alveolar macrophages (Fig. 2a and Supplementary Fig. S6; Supplementary Table S1). There was also labeling in a subset of glomerular cells (Supplementary Fig. S6). *Mlkl* transcripts were identified in many cell types, including Kupffer cells and certain glomerular cells, but labeling was frequently inconsistent with subpopulations containing 1-2 spots per cell (Fig. 2a, b and Supplementary Figs. S6–S8; Supplementary Table S1).

RIPK1, RIPK3, and *Mlkl* were expressed in barrier epithelium (small and large intestine, glandular stomach, gallbladder, corneal epithelium, conjunctiva, urothelium, endometrium, and stratified squamous epithelia) (Fig. 2b and Supplementary Figs. S6 and 7; Supplementary Table S1). Variable labeling was observed in ductal epithelium, including pancreatic and salivary ducts (Fig. 2 and Supplementary Fig. S6). There was segmental RIPK1, RIPK3, and *Mlkl* expression in the epididymis, with inconsistent expression levels of the molecules between segments (Supplementary Fig. S7a). There was rare to no RIPK3 labeling in bronchioles, pancreatic acinar cells, hepatocytes, or adrenal epithelial cells (Fig. 2a, b, and Supplementary Fig. S6; Supplementary Table S1). Rare RIPK3 labeling in renal tubules was primarily in the medulla (Fig. 2b). It was difficult to distinguish specific RIPK1 labeling in some tissues including the pancreas, kidney, adrenal, salivary gland, and liver (Fig. 2a, b and Supplementary Fig. S6; Supplementary Table S1). Nonetheless, most cell types had detectable *Ripk1* by ISH, including renal tubular epithelium, pancreatic acinar cells, and hepatocytes (Fig. 2c and Supplementary Fig. S1). The strongest *Ripk1* labeling was in the small intestinal epithelium, thymus, and splenic white pulp (Figs. 1b, 2c, and Supplementary Fig. S1).

In the adrenal, RIPK1 expression was highest in the zona glomerulosa, whereas labeling was weaker in the medulla (Supplementary Fig. S6). In the brain, RIPK1 was detected in endothelial cells and the meninges, with weak labeling in the choroid plexus. Occasional glial and ependymal cells had labeling near the limits of detection (Supplementary Fig. S6). RIPK3 was also detected in endothelial cells in the brain, with the strongest signal in the choroid plexus endothelium (Supplementary Fig. S6). *Mlkl* was detected in the meninges and choroid plexus, with rare labeling in the neuroparenchyma or other nervous tissues (Supplementary Fig. S6; Supplementary Table S1). Other significant labeling of the neuroparenchyma, including labeling of neurons, was not evident (Supplementary Fig. S6; Supplementary Table S1).

In the testis, RIPK3 labeling was strongest in a basal cell population, presumably germ cells, in seminiferous tubules, whereas RIPK1 and *Mlkl* labeling was predominately in the interstitium (Supplementary Fig. S7a; Supplementary Table S1). Increased RIPK1 and RIPK3 labeling was associated with degenerate tubules (Supplementary Fig. S7b). RIPK1 and *Mlkl* were expressed throughout the ovary, whereas RIPK3 labeling was strong in vasculature, but heterogeneous in granulosa, thecal, and luteal cells (Supplementary Fig. S7a; Supplementary Table S1).

### Tissue expression patterns of ZBP1

**Fig. 3:**
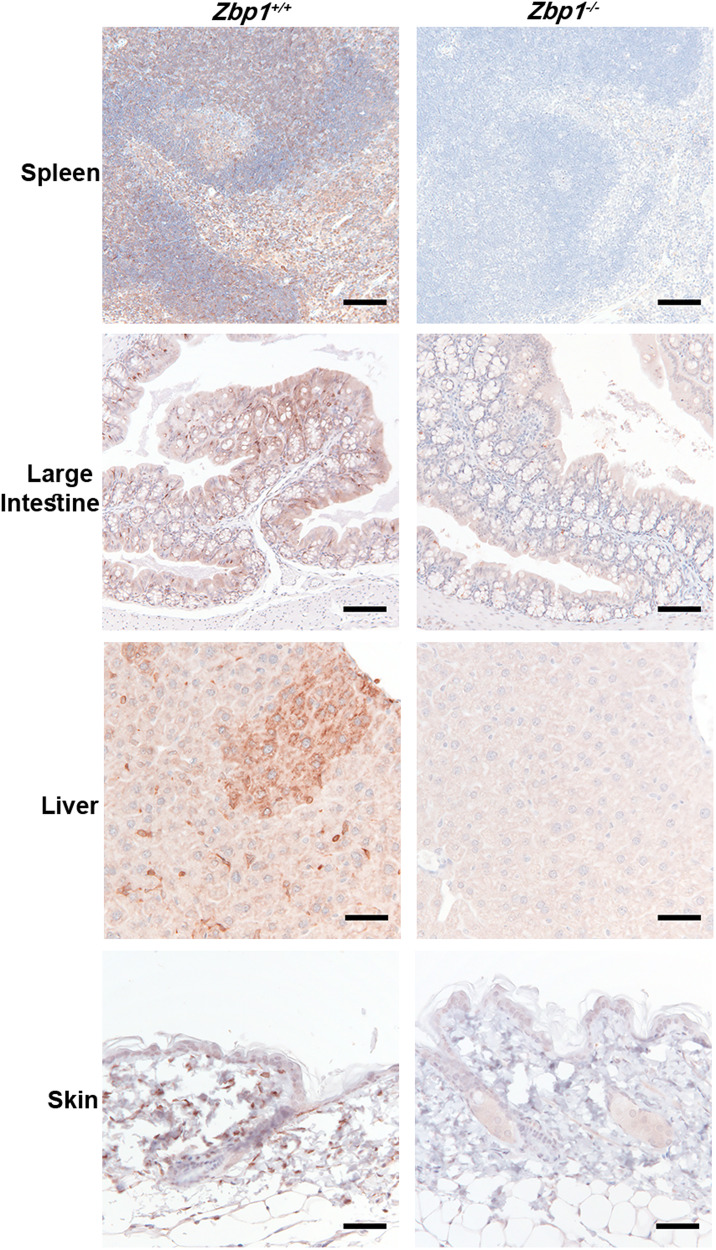
ZBP1 IHC in mouse tissues. ZBP1 IHC in mouse tissues. Scale bars, 100 μm (spleen, large intestine) or 50 µm (liver, skin). Results representative of 5 WT and 4 *Zbp1*^*−/−*^ mice.

ZBP1 expression was most prominent and consistent in interstitial cells, including Kupffer cells and macrophages in lymphoid tissues (Figs. 3, Supplementary Figs. S4, and S9; Supplementary Table S1). ZBP1 was detected in lymphocytes, particularly in splenic periarteriolar lymphoid sheaths and the lymph node paracortex. There was inconsistent labeling in lymphoid follicles, with weaker ZBP1 labeling in germinal centers (Fig. 3 and Supplementary Fig. S4; Supplementary Table S1). Endothelium had occasional labeling (Supplementary Fig. S4; Supplementary Table S1).

Small intestinal epithelium had diffuse villous labeling, with reduced labeling in the upper crypts (Fig. 1e). Large intestinal labeling was heterogeneous in superficial enterocytes with stronger labeling in suspected intraepithelial lymphocytes (Fig. 3). Focal to segmental epithelial labeling was observed in the tongue, bronchioles, salivary ducts, conjunctiva, stomach, epididymis, and rare renal tubules. ZBP1 was largely undetected in the epidermis, urothelium of the bladder, pancreatic acinar cells, and adrenal epithelial cells (Fig. 3 and Supplementary Fig. S4; Supplementary Table S1). The liver contained multifocally increased hepatocyte labeling, suggesting regionally increased expression (Fig. 3). In the brain, ZBP1 was primarily detected in individual immune cells in the meninges and choroid plexus. Few vessels also had labeling, but neuronal labeling was not evident (Supplementary Fig. S4). In the uterus, ZBP1 was expressed in the endometrial epithelium and stroma. Individual cells expressed ZBP1 in the interstitium of the testis and ovary (Supplementary Fig. S4; Supplementary Table S1).

### Elevated expression of ZBP1 in *Casp8*^*−/−*^*Tnfr1*^*−/−*^ embryos

**Fig. 4:**
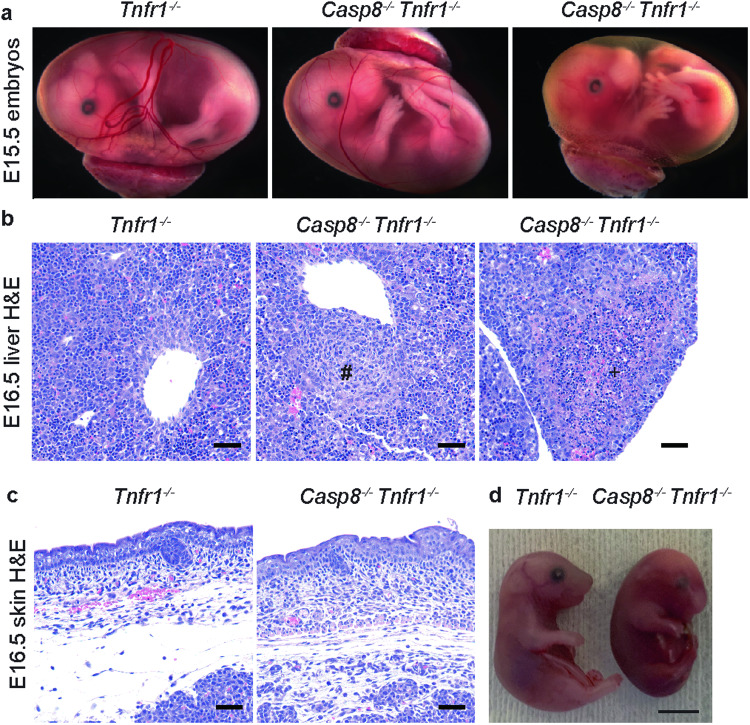
*Casp8*^*−/−*^*Tnfr1*^*−/−*^ embryos have skin and liver defects. **a** E15.5 embryos. *Casp8*^*−/−*^ *Tnfr1*^−/−^ embryos from 4 different litters appeared grossly normal (*n* = 4, middle panel) or lacked normal yolk sac vasculature (*n* = 1, right panel). A representative *Tnfr1*^*−/−*^ littermate is shown (*n* = 5; left panel). **b** E16.5 liver sections stained with hematoxylin and eosin (H&E). Scale bars, 50 μm. Myeloid aggregates (#) were observed in all *Casp8*^*−/−*^ *Tnfr1*^−/−^ embryos (*n* = 5). Foci of hepatocellular death (+) were observed in 3 out of 5 *Casp8*^*−/−*^ *Tnfr1*^−/−^ embryos. A representative section from a *Tnfr1*^*−/−*^ littermate (*n* = 3) is shown. **c** Representative H&E-stained skin sections from the E16.5 embryos in **b**. Scale bars, 50 μm. **d** E18.5 embryos. Results representative of 4 *Tnfr1*^*−/−*^ and 5 *Casp8*^*−/−*^ *Tnfr1*^−/−^ embryos.

ZBP1-driven necroptosis contributes to the death of *Casp8*^*−/−*^ *Ripk1*^*−/−*^ mice at birth [14], and TNFR1 and ZBP1 contribute to ileitis and colitis in mice lacking caspase-8 in intestinal epithelial cells [26]. We investigated ZBP1 expression during mouse embryogenesis and its potential contribution to the embryonic lethality of *Casp8*^*−/−*^ *Tnfr1*^*−/−*^ mice. Consistent with published results [19], TNFR1 deficiency permitted *Casp8*^*−/−*^ embryos to survive beyond E11. Four out of 5 *Casp8*^*−/−*^ *Tnfr1*^*−/−*^ embryos appeared grossly normal at E15.5, although aberrant yolk sac vasculature was observed in one embryo (Fig. 4a). At E16.5, all *Casp8*^*−/−*^ *Tnfr1*^*−/−*^ fetal livers contained aggregates of macrophages and, in a subset (3 out of 5), there was evidence of increased cell death in the form of necrotic foci and karyorrhectic debris (Fig. 4b). Four out of 5 *Casp8*^*−/−*^ *Tnfr1*^*−/−*^ embryos also had skin lesions, with increased dermal leukocytes and/or subcutaneous edema (Fig. 4c). By E18.5, all *Casp8*^*−/−*^ *Tnfr1*^*−/−*^ embryos had died (Fig. 4d and Table 1).

**Table 1:** ZBP1 and TRIF contribute to embryonic lethality of *Casp8*^*−/−*^ *Tnfr1*^*−/−*^ mice.

Age	Genetic background	*Casp8*^*+/+*^	*Casp8*^*+/-*^	*Casp8*^*−/−*^
E18.5	*Tnfr1*^*−/−*^	4	18	5 (all dead)
	*Tnfr1*^*−/−*^ *Trif*^*−/−*^	5	15	6
	*Tnfr1*^*−/−*^ *Zbp1*^*−/−*^	10	25	17
	*Trnfr1*^*−/−*^ *Trif*^*−/−*^ *Zbp1*^*−/−*^	8	13	7
P0	*Tnfr1*^*−/−*^ *Trif*^*−/−*^	8	8	0
P4-7	*Tnfr1*^*−/−*^	21	45	0
	*Tnfr1*^*−/−*^ *Trif*^*−/−*^	61	137	0
	*Tnfr1*^*−/−*^ *Zbp1*^*−/−*^	88	156	9
	*Trnfr1*^*−/−*^ *Trif*^*−/−*^ *Zbp1*^*−/−*^	65	94	41

Offspring numbers from intercrossing Casp8^+/-^ mice.

**Fig. 5:**
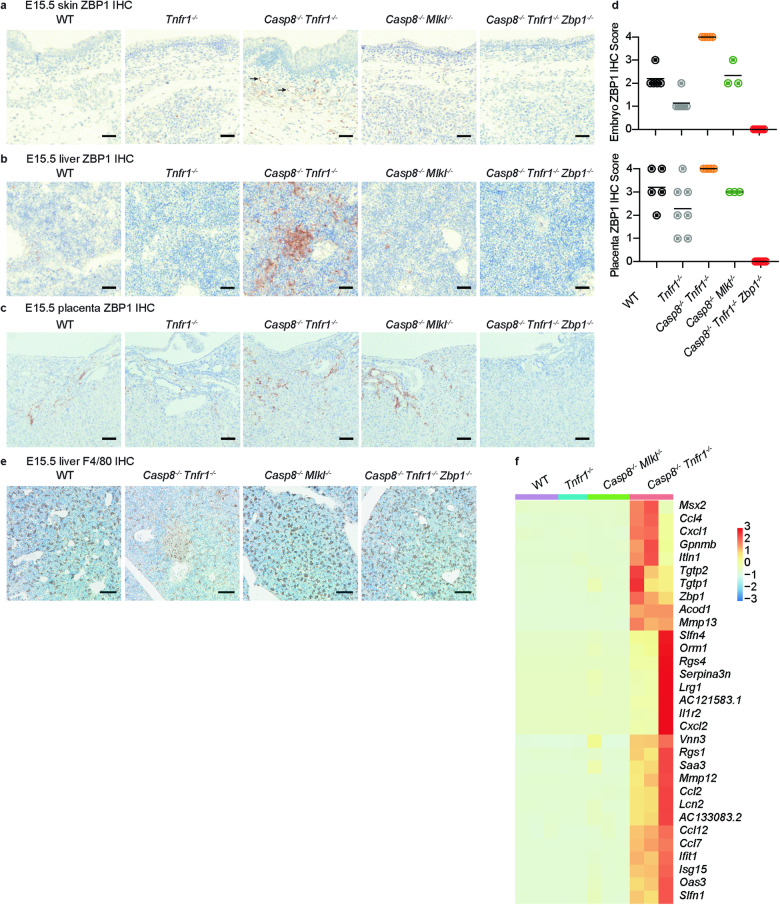
ZBP1 is expressed in myeloid cells and the vasculature of E15.5 *Casp8*^*−/−*^*Tnfr1*^*−/−*^ embryos. E15.5 skin (**a**), liver (**b**), and placenta (**c**) sections with ZBP1 immunolabeling (brown). Scale bars, 50 μm (**a**, **b**) or 100 μm (**c**). Images representative of WT (*n* = 5), *Tnfr1*^*−/−*^ (*n* = 7), *Casp8*^*−/−*^ *Tnfr1*^*−/−*^ (*n* = 4), *Casp8*^*−/−*^ *Mlkl*^*−/−*^ (*n* = 3), and *Casp8*^*−/−*^ *Tnfr1*^*−/−*^ *Zbp1*^*−/−*^ (*n* = 7) embryos. Arrows highlight vascular labeling in *Casp8*^*−/−*^ *Tnfr1*^*−/−*^ skin. **d** ZBP1 IHC scores for the embryos in **a**–**c** and their placentas. Lines indicate the mean. See methods for scoring criteria. **e** E15.5 liver sections with immunolabeling of F4/80 (brown). Scale bars, 100 μm. Images representative of WT (*n* = 5), *Casp8*^*−/−*^ *Tnfr1*^*−/−*^ (*n* = 5), *Casp8*^*−/−*^ *Tnfr1*^*−/−*^ *Zbp1*^*−/−*^ (*n* = 7), and *Casp8*^*−/−*^ *Mlkl*^*−/−*^ (*n* = 3) embryos. **f** Heatmap shows differentially expressed genes in E15.5 *Casp8*^*−/−*^ *Tnfr1*^*−/−*^ fetal livers (*n* = 3) when compared with WT (*n* = 3), *Tnfr1*^*−/−*^ (*n* = 2), and *Casp8*^*−/−*^ *Mlkl*^*−/−*^ (*n* = 3) fetal livers.

At E15.5, ZBP1 IHC labeled low numbers of cells throughout WT embryos (Fig. 5a, b). These ZBP1^+^ cells had round to stellate morphologies, and probably represented macrophage lineages. Foci of cells and occasional vessels in the placental labyrinth also expressed ZBP1 (Fig. 5c). *Casp8*^*−/−*^ *Tnfr1*^*−/−*^ embryos had more ZBP1^+^ cells than WT or *Tnfr1*^*−/−*^ embryos, with myeloid infiltrates in the liver and vessels being labeled (Fig. 5a, b, d). Myeloid aggregates in the *Casp8*^*−/−*^ *Tnfr1*^*−/−*^ liver included F4/80^+^ macrophages (Fig. 5e). No ZBP1^+^ cells were detected in *Casp8*^*−/−*^ *Tnfr1*^*−/−*^ *Zbp1*^*−/−*^ embryos or their placentas, confirming the specificity of the anti-ZBP1 antibody (Fig. 5a–d). Cells expressing RIPK1, RIPK3, or *Mlkl* were not markedly increased in E15.5 *Casp8*^*−/−*^ *Tnfr1*^*−/−*^ skin (Supplementary Fig. S10a). Interestingly, expression of ZBP1 in *Casp8*^*−/−*^ *Mlkl*^*−/−*^ embryos was comparable to that in WT embryos. This result could indicate that necroptosis in *Casp8*^*−/−*^ *Tnfr1*^*−/−*^ embryos amplified the number of ZBP1^+^ cells. However, we cannot exclude that TNFR1 signaling in *Casp8*^*−/−*^ embryos somehow restricts ZBP1 expression independent of necroptosis. Distinguishing between these two possibilities awaits the generation of *Casp8*^*−/−*^ *Tnfr1*^*−/−*^ *Mlkl*^*−/−*^ mice.

Given that *Zbp1* is an interferon-stimulated gene (ISG) [32], we used RNA sequencing to determine if other ISGs were elevated in E15.5 *Casp8*^*−/−*^ *Tnfr1*^*−/−*^ fetal livers (Fig. 5f). Compared to WT, *Tnfr1*^*−/−*^, and *Casp8*^*−/−*^ *Mlkl*^*−/−*^ livers, *Casp8*^*−/−*^ *Tnfr1*^*−/−*^ livers demonstrated significantly elevated expression of several genes, including the ISGs *Zbp1*, *Ifit1*, *Isg15*, *Oas3*, and *Slfn1* [33–35]. Therefore, *Zbp1* is not the only ISG that is upregulated in *Casp8*^*−/−*^ *Tnfr1*^*−/−*^ embryos.

Next, we determined whether ZBP1 and/or TRIF caused lethality in *Casp8*^*−/−*^ *Tnfr1*^*−/−*^ embryos. While *Casp8*^*−/−*^ *Tnfr1*^*−/−*^ *Trif*^*−/−*^ embryos were observed at the expected frequency at E18.5, they were often autolyzed and no *Casp8*^*−/−*^ *Tnfr1*^*−/−*^ *Trif*^*−/−*^ pups were found at birth (post-natal day 0 [P0]; Table 1). By contrast, rare *Casp8*^*−/−*^ *Tnfr1*^*−/−*^ *Zbp1*^*−/−*^ pups survived the perinatal period (4% identified between P4 and P7 versus the expected 25%) (Table 1). Thus, ZBP1 contributes to lethality in *Casp8*^*−/−*^ *Tnfr1*^*−/−*^ embryos.

**Fig. 6:**
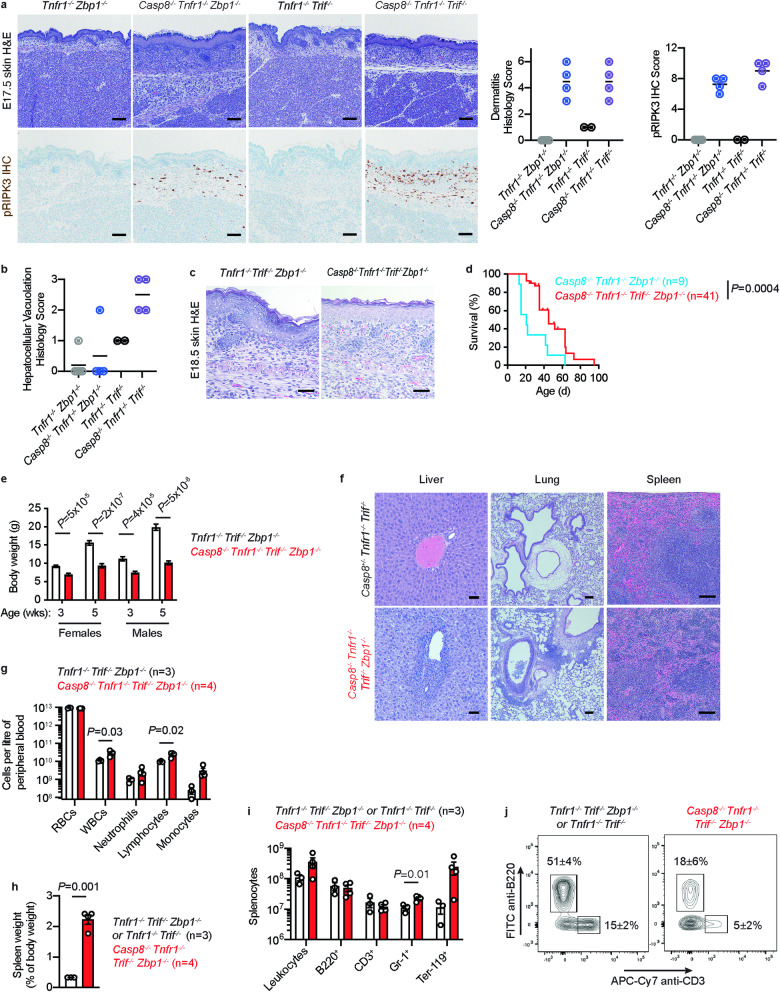
Combined loss of ZBP1 and TRIF delays lethality in *Casp8*^*−/−*^*Tnfr1*^*−/−*^ mice. **a** E17.5 skin sections stained with H&E or immunolabeled with phospho-RIPK3 T^231^, S^232^ (pRIPK3, brown). Scale bars, 100 μm. Graphs show dermatitis histology scores and pRIPK3 IHC scores for *Tnfr1*^*−/−*^ *Zbp1*^*−/−*^ (*n* = 5), *Casp8*^*−/−*^ *Tnfr1*^*−/−*^ *Zbp1*^*−/−*^ (*n* = 4), *Tnfr1*^*−/−*^ *Trif*^*−/−*^ (*n* = 2), and *Casp8*^*−/−*^ *Tnfr1*^*−/−*^ *Trif*^*−/−*^ (*n* = 4) embryos. Lines indicate the mean. **b** Hepatocellular vacuolation histology scores for the embryos in **a**. Lines indicate the mean. See methods for scoring criteria used in **a** and **b**. **c** Representative H&E-stained skin sections from E18.5 *Casp8*^*−/−*^ *Tnfr1*^*−/−*^ *Trif*^*−/−*^ *Zbp1*^*−/−*^ (*n* = 3) and *Tnfr1*^*−/−*^ *Trif*^*−/−*^ *Zbp1*^*−/−*^ (*n* = 3) embryos demonstrating epidermal thickening and increased dermal cellularity in the *Casp8*^*−/−*^ *Tnfr1*^*−/−*^ *Trif*^*−/−*^ *Zbp1*^*−/−*^embryo. Scale bar, 50 μm. **d** Kaplan–Meier curves of mouse survival. *P*-value determined by 2-sided log-rank test. **e** Body weights of *Casp8*^*−/−*^ *Tnfr1*^*−/−*^ *Trif*^*−/−*^ *Zbp1*^*−/−*^ (red bars; females, *n* = 17 aged 3 weeks, *n* = 12 aged 5 weeks; males, *n* = 11 aged 3 wks, *n* = 10 aged 5 weeks) and *Tnfr1*^*−/−*^ *Trif*^*−/−*^ *Zbp1*^*−/−*^ (white bars; females, *n* = 14 aged 3 weeks, *n* = 11 aged 5 weeks; males, *n* = 13 aged 3 wks, *n* = 11 aged 5 weeks) mice. Bars indicate the mean ± s.e.m. *P*-values determined by 2-sided student t-test with Welch’s correction. **f** Representative liver, lung, and spleen sections from male *Casp8*^*−/−*^ *Tnfr1*^*−/−*^ *Trif*^*−/−*^ *Zbp1*^*−/−*^ mice (*n* = 3) with increased cellular infiltrates in the lung and liver and increased splenic hematopoiesis and male littermate controls (*Casp8*^*+/-*^ *Tnfr1*^*−/−*^ *Trif*^*−/−*^, *n* = 2 and *Casp8*^*+/-*^ *Tnfr1*^*−/−*^ *Trif*^*+/-*^ *Zbp1*^*+/-*^, *n* = 1) aged 9-10 weeks. Scale bars, 100 μm (lung, spleen) or 50 μm (liver). **g** Peripheral blood cell counts in *Casp8*^*−/−*^ *Tnfr1*^*−/−*^ *Trif*^*−/−*^ *Zbp1*^*−/−*^ (*n* = 4) and *Tnfr1*^*−/−*^ *Trif*^*−/−*^ *Zbp1*^*−/−*^ (*n* = 4) mice aged 4 weeks. Bars indicate the mea*n* ± s.e.m. *P*-values are shown if *P* < 0.05 by 2-sided t-test with Welch’s correction. **h** Spleen weight as a percentage of body weight for mice aged 4-5 weeks. Red bars, *Casp8*^*−/−*^ *Tnfr1*^*−/−*^ *Trif*^*−/−*^ *Zbp1*^*−/−*^ mice (*n* = 4; 1 male, 3 females). White bars, littermate *Tnfr1*^*−/−*^ *Trif*^*−/−*^ *Zbp1*^*−/−*^ (*n* = 2; 1 male, 1 female) or *Tnfr1*^*−/−*^ *Trif*^*−/−*^ (*n* = 1, female) mice. Bars indicate the mean ± s.e.m. *P*-value determined by 2-sided student t-test with Welch’s correction. **i** Splenic leukocyte subsets for the mice in **h**. Bars indicate the mean ± s.e.m. *P*-values are shown if *P* < 0.05 by 2-sided t-test with Welch’s correction. **j** Representative spleen flow cytometry contour plots of the mice in **h**.

At E17.5, both *Casp8*^*−/−*^ *Tnfr1*^*−/−*^ *Trif*^*−/−*^ and *Casp8*^*−/−*^ *Tnfr1*^*−/−*^ *Zbp1*^*−/−*^ embryos had dermatitis that primarily affected the dorsal skin (Fig. 6a). Dermal cellular infiltrates expressed RIPK1, RIPK3, and *Mlkl* and contained phospho-RIPK3 T^231^, S^232^, a hallmark of necroptosis signaling [36] (Fig. 6a and Supplementary Fig. S10b). *Casp8*^*−/−*^ *Tnfr1*^*−/−*^ *Trif*^*−/−*^ embryos also had decreased hepatocellular vacuolation (loss of glycogen and/or lipid), suggestive of metabolic imbalance (Fig. 6b). TRIF nevertheless contributed to perinatal lethality in *Casp8*^*−/−*^ *Tnfr1*^*−/−*^ *Zbp1*^*−/−*^ mice because most *Casp8*^*−/−*^ *Tnfr1*^*−/−*^ *Trif*^*−/−*^ *Zbp1*^*−/−*^ pups survived the perinatal period (21% identified between P4 and P7 versus the expected 25%) (Table 1). Interestingly, E18.5 *Casp8*^*−/−*^ *Tnfr1*^*−/−*^ *Trif*^*−/−*^ *Zbp1*^*−/−*^ embryos had a thickened epidermis and increased dermal cellularity when compared to *Tnfr1*^*−/−*^ *Trif*^*−/−*^ *Zbp1*^*−/−*^ littermates (Fig. 6c). Skin lesions can be triggered by TRAIL-, FAS ligand- or TNF-induced keratinocyte cell death [37], so the skin phenotype of *Casp8*^*−/−*^ *Tnfr1*^*−/−*^ *Trif*^*−/−*^ *Zbp1*^*−/−*^ embryos might reflect necroptosis induced by death receptors other than TNFR1.

In contrast to *Casp8*^*−/−*^ *Ripk1*^*−/−*^ *Trif*^*−/−*^ *Zbp1*^*−/−*^ mice, which largely survive to adulthood but then develop lymphadenopathy that is characteristic of caspase-8 deficiency [14], *Casp8*^*−/−*^ *Tnfr1*^*−/−*^ *Trif*^*−/−*^ *Zbp1*^*−/−*^ pups exhibited stunted growth and were typically euthanized owing to ill health between 5 and 7 weeks of age (Fig. 6d, e). Inflammatory infiltrates were observed in most adult *Casp8*^*−/−*^ *Tnfr1*^*−/−*^ *Trif*^*−/−*^ *Zbp1*^*−/−*^ tissues, including the lung, liver, and spleen (Fig. 6f). *Casp8*^*−/−*^ *Tnfr1*^*−/−*^ *Trif*^*−/−*^ *Zbp1*^*−/−*^ peripheral blood also contained more white blood cells than *Tnfr1*^*−/−*^ *Trif*^*−/−*^ *Zbp1*^*−/−*^ blood (Fig. 6g). Splenomegaly in *Casp8*^*−/−*^ *Tnfr1*^*−/−*^ *Trif*^*−/−*^ *Zbp1*^*−/−*^ mice (Fig. 6h) reflected extramedullary hematopoiesis rather than an accumulation of B220^+^CD3^+^ T cells (Fig. 6f, i, j), the latter underlying splenomegaly and lymphadenopathy in older *Casp8*^*−/−*^ *Ripk3*^*−/−*^ or *Casp8*^*−/−*^ *Mlkl*^*−/−*^ mice [22]. Collectively, these data point to *Casp8*^*−/−*^ *Tnfr1*^*−/−*^ *Trif*^*−/−*^ *Zbp1*^*−/−*^ mice having an autoinflammatory phenotype, which we infer from the improved survival of *Casp8*^*−/−*^ *Ripk1*^*−/−*^ *Trif*^*−/−*^ *Zbp1*^*−/−*^ mice [14], is due to aberrant RIPK1-dependent necroptosis. Death receptors other than TNFR1 that can engage RIPK1-dependent necroptosis include DR3, FAS, and TRAIL [3, 38].

## Discussion

We show that RIPK1, RIPK3, and *Mlkl* are expressed in immune cells, endothelial cells, and a subset of epithelial cells. Epithelial expression was most pronounced in the small and large intestines, but was also present in other barrier epithelia. By contrast, expression of these molecules was either weak or below detection limits in non-mucosal epithelium including in the pancreas, kidney, liver, and nervous tissue. The expression of RIPK1, RIPK3, and *Mlkl* in immune and endothelial cells is consistent with experiments investigating lethality in *Casp8*^*−/−*^ and *Ripk1*^*−/−*^ mice. Vascular collapse and embryonic mortality owing to caspase-8 deficiency is prevented by loss of RIPK1, RIPK3, or MLKL [5, 6, 19–22, 24]. RIPK3 also drives necroptosis in hematopoietic cells lacking RIPK1 [39]. Our tissue atlas provides additional nuance to previous observations, such as the unique increase in expression of RIPK3 in germinal centers. *Ripk3*^*−/−*^ mice are viable [40] and the role of RIPK3 in germinal centers is unknown.

Epithelial expression of RIPK1, RIPK3, and *Mlkl* alludes to their role in innate immunity. Expression of all three is highest in the intestinal epithelium, which continuously interacts with microbiota. Other epithelial labeling was strongest in barrier tissues including the skin, uterus, tongue, and urinary bladder. By contrast, parenchymal organs such as the liver, kidney, and pancreas had limited epithelial expression, although low level expression of *Ripk1* in hepatocytes appears to limit TNF-induced apoptosis [41]. We failed to detect robust expression of RIPK1, RIPK3, or *Mlkl* in the neuroparenchyma of the brain, which is consistent with reports that neither RIPK3 nor MLKL can be detected in the mouse central nervous system by western blotting [42–44]. A previous study suggested RIPK3 expression in cultured mouse neurons using an immunofluorescence assay, but lacked *Ripk3*^*−/−*^ neurons as negative controls [45].

ZBP1 was expressed in immune cells with heterogeneous expression in epithelial cells. This heterogeneous labeling was particularly noted in the liver and tongue. The small intestine also had a distinct absence of labeling in the upper crypts, but strong expression in the base of crypts and villi. *Zbp1* is an ISG [32], so heterogeneous expression could indicate localized differences in interferon signaling. *Mlkl* can also be induced by interferon [19], but had a more consistent pattern of expression. Differences in the regulation of these genes requires further investigation. ZBP1 IHC revealed elevated numbers of ZBP1^+^ cells in *Casp8*^*−/−*^ *Tnfr1*^*−/−*^ embryos compared to WT or *Casp8*^*−/−*^ *Mlkl*^*−/−*^ embryos. The latter result suggests that necroptosis can induce ZBP1 expression and/or promote the expansion of ZBP1^+^ cells. ZBP1 contributed to the death of *Casp8*^*−/−*^ *Tnfr1*^*−/−*^ embryos because loss of *Zbp1* prolonged their survival, and combined loss of *Zbp1* and *Trif* even more so. Whether ZBP1 uses its Zα domains to sense endogenous Z-form nucleic acids in this setting is unclear. TRIF contributed to the perinatal lethality of *Casp8*^*−/−*^ *Tnfr1*^*−/−*^ *Zbp1*^*−/−*^ mice, but whether TRIF is inducing necroptosis in the same cell types as ZBP1 is unclear. A suitable TRIF IHC assay has proven elusive to date, but *Trif* ISH may prove informative in the future.

## Materials and methods

### Mice

Studies complied with relevant ethics regulations and were approved by the Genentech institutional animal care and use committee. *Casp8*^*+/-*^ [29], *Mlkl*^*−/−*^ [31], *Ripk1*^*+/-*^ [29], *Ripk3*^*−/−*^ [29], *Ripk3*^*D161N/+*^ [29], *Tnfr1*^*−/−*^ [46], *Trif*^*−/−*^ [47], and *Zbp1*^−/−^ [14] mice were maintained on a C57BL/6 N genetic background. *Casp8*^*+/-*^, *Casp8*^*C362A/+*^, *Ripk1*^*+/-*^, *Ripk3*^*D161N/+*^, and *Zbp1*^−/−^ mice were generated using C57BL/6 N ES cells, whereas *Mlkl*^*−/−*^, *Ripk3*^*−/−*^, *Tnfr1*^*−/−*^, and *Trif*^*−/−*^ mice were backcrossed to C57BL/6 N for at least 11 generations. Embryos were designated E0.5 on the morning a vaginal plug was detected. Newborn mice were designated P0 on their day of birth.

### Histology

Formalin-fixed, paraffin-embedded (FFPE) 4-µm embryo sections were stained with hematoxylin and eosin (StatLab, McKinney, Texas), and evaluated blinded as to genotype. Skin was scored for epidermal hyperplasia (0, normal undulating epithelium with 1–3 cell thick stratum spinosum; 1, partial or complete loss of undulation with 3–4 cell thick stratum spinosum; 2, 5–6 cell thick spinosum; or 3, >6 cell thick spinosum), inflammatory infiltrates (0, within normal limits; 1, mildly increased cellularity without significant epidermal expansion; 2, increased dermal cellularity with < 2× dermal expansion; or 3, densely increased dermal cellularity with >2× dermal expansion), and brown adipose tissue atrophy (0, within normal limits; 1, focal area of brown adipose tissue loss; 2, multifocal atrophy of brown adipose tissue; or 3, locally extensive loss/atrophy of brown adipose tissue). Dermatitis scores were the sum of these three scores. Liver was scored for hepatocellular vacuolation (0, diffuse hepatocellular vacuolation; 1, vacuolation is mildly decreased, but present in most to all hepatocytes; 2, regional loss of hepatocellular vacuolation; or 3, diffuse loss of hepatocellular vacuolation).

### IHC and ISH

Adult mouse tissues were from mice aged 9–11 weeks. FFPE 4-µm sections were deparaffinized in xylenes and rehydrated through graded alcohols to distilled water. IHC labeling of pRIPK3 T^231^, S^232^ and F4/80 were described previously [48, 49]. pRIPK3 labeling in the skin, liver, lung, and intestines was scored blindly: (0) no labeling, (1) 1–10 individual positive cells/organ cross-section, (2) > 10 individual positive cells/organ cross-section, (3) individual and aggregates of labeled cells, or (4) extensive labeling. The final score was the sum of the individual tissue scores.

RIPK1, RIPK3, and ZBP1 IHC was performed with 3.5 µg/ml 10C7 rat anti-mouse RIPK1 (Genentech, South San Francisco, CA, USA), 2 µg/ml rabbit anti-RIPK3 polyclonal (Abcam, ab62344), 7.5 μg/ml 1G6 rat anti-RIPK3 (Genentech), and 7.5 µg/ml GN58.3 rat anti-mouse ZBP1 (Genentech). Full-length *Mlkl* and *Ripk1* ISH was performed with 20zz RNAscope probes targeting *Mlkl* base pairs 737–1699 or *Ripk1* base pairs 267–1218 (Advanced Cell Diagnostics [ACD], Newark, CA, USA), respectively. *Mlkl* exon 3 was detected using a 1zz Basescope probe targeting base pairs 816–864 (ACD). Full details of the IHC and ISH methods are provided in the Supplementary Materials.

ZBP1 labeling in embryos was scored as: (0) no labeling, (1) rare individual labeled cells, frequently difficult to identify, (2) individual labeled cells distributed throughout most tissues, (3) in addition to individual labeled cells, occasional distinct aggregates of immunolabeled cells (besides in the thymus), or (4) increased individual labeled cells and extensive immunolabeled infiltrates in the liver. Labeling in the placenta was scored as: (0) no labeling, (1) mild, multifocal labeling in the labyrinth characterized by few relatively small foci of labeled cells, (2) few, moderately sized labeled foci in the labyrinth, (3) moderate, multifocal labeled foci in the labyrinth, or (4) locally extensive labeling in the labyrinth.

### Flow cytometry

Splenocytes were labeled with APC-Cy7-anti-CD3 (557596), BV421-anti-Gr-1 (562709), FITC-anti-B220 (553088), and PE-Cy7-anti-TER-119 (557915) antibodies (BD Biosciences, San Jose, CA, USA) in 2% normal rat serum and 1 μg/ml 2.4G2 anti-CD16/CD32 (BD Biosciences, 553142). Dead cells that stained with 7-AAD (BD Biosciences), plus doublets, identified by their FSC-A versus FSC-W profiles, were excluded from analyses as described [50]. Data were acquired using a BD FACSCantoII (BD Biosciences) cytometer and BD FACSDiva 9.0. Data were analyzed with FlowJo 10.7.1.

### RNA sequencing

Total RNA isolated from E15.5 fetal livers was quantified with a Qubit RNA HS Assay Kit (Thermo Fisher Scientific, Waltham, MA, USA). Quality was assessed using RNA ScreenTape on TapeStation 4200 (Agilent). For sequencing library generation, the Truseq Stranded mRNA kit (Illumina, San Diego, CA, USA) was used with an input of 100–1000 nanograms of total RNA. Libraries were quantified with a Qubit dsDNA HS Assay Kit (Thermo Fisher Scientific) and the average library size was determined using D1000 ScreenTape on TapeStation 4200 (Agilent Technologies). Libraries were pooled and sequenced on NovaSeq 6000 (Illumina) to generate 30 million single-end 50-bp reads for each sample. Raw FASTQ reads were aligned to the mouse genome (GRCm38/mm10) using GSNAP (version 2013-11-10) [51] with parameters “-M 2 -n 10 -B 2 -i 1 -N 1 -w 200000 -E 1 --pairmax-rna=200000 --clip-overlap”. Differential expression analysis was performed on uniquely mapped reads with Limma [52]. Genes with log2 fold change >4 and adjusted *p*-value < 0.001 across all comparisons were included in the heatmap.

### Western blots

Tissue lysates were prepared in 20 mM Tris.HCl pH 7.5, 135 mM NaCl, 1.5 mM MgCl_2_, 1 mM EGTA, 1% Triton X-100, 10% glycerol, and 2× Halt protease inhibitor cocktail (Thermo Fisher Scientific, Waltham, MA, USA). GN58.3 anti-mouse ZBP1 (Genentech) was raised against ZBP1 residues A2-I153. Protein loading was assessed by detecting GAPDH (8884, Cell Signaling Technology).

### Statistics

No sample size calculations were performed. There was no method of randomization. No samples or animals were excluded from analyses. Statistics were calculated using Prism 9.1.2.

### Reporting summary

Further information on research design is available in the Nature Research Reporting Summary linked to this article.

### Supplementary information

Supplementary Figures

Supplementary Methods

Table S1

Reporting Summary

## Data Availability

Mice and antibody reagents generated by Genentech are available under a material transfer agreement with Genentech. RNAseq data has been deposited to GEO (accession # GSE254754).
