# Supplementary material for: ZBP1 and TRIF trigger lethal necroptosis in mice lacking caspase-8 and TNFR1
Source: Cell Death Differ. 2024 Mar 28;31(5):672–82. doi: 10.1038/s41418-024-01286-6 (PMC11093969; doi:10.1038/s41418-024-01286-6)
Supplement: Supplementary file 1 — Supplementary Figures [file 41418_2024_1286_MOESM1_ESM.pdf]

Supplementary Figures

ZBP1 and TRIF trigger lethal necroptosis in mice lacking caspase-8 and TNFR1

Solon et al.

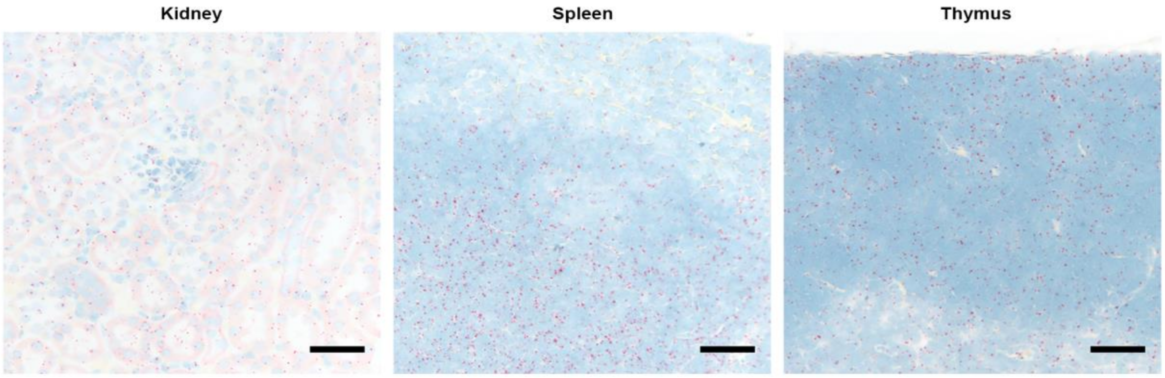

Figure S1. *Ripk1* ISH in WT kidney, spleen, and thymus. Scale bars, 50 µm. Results representative of 5 WT mice.

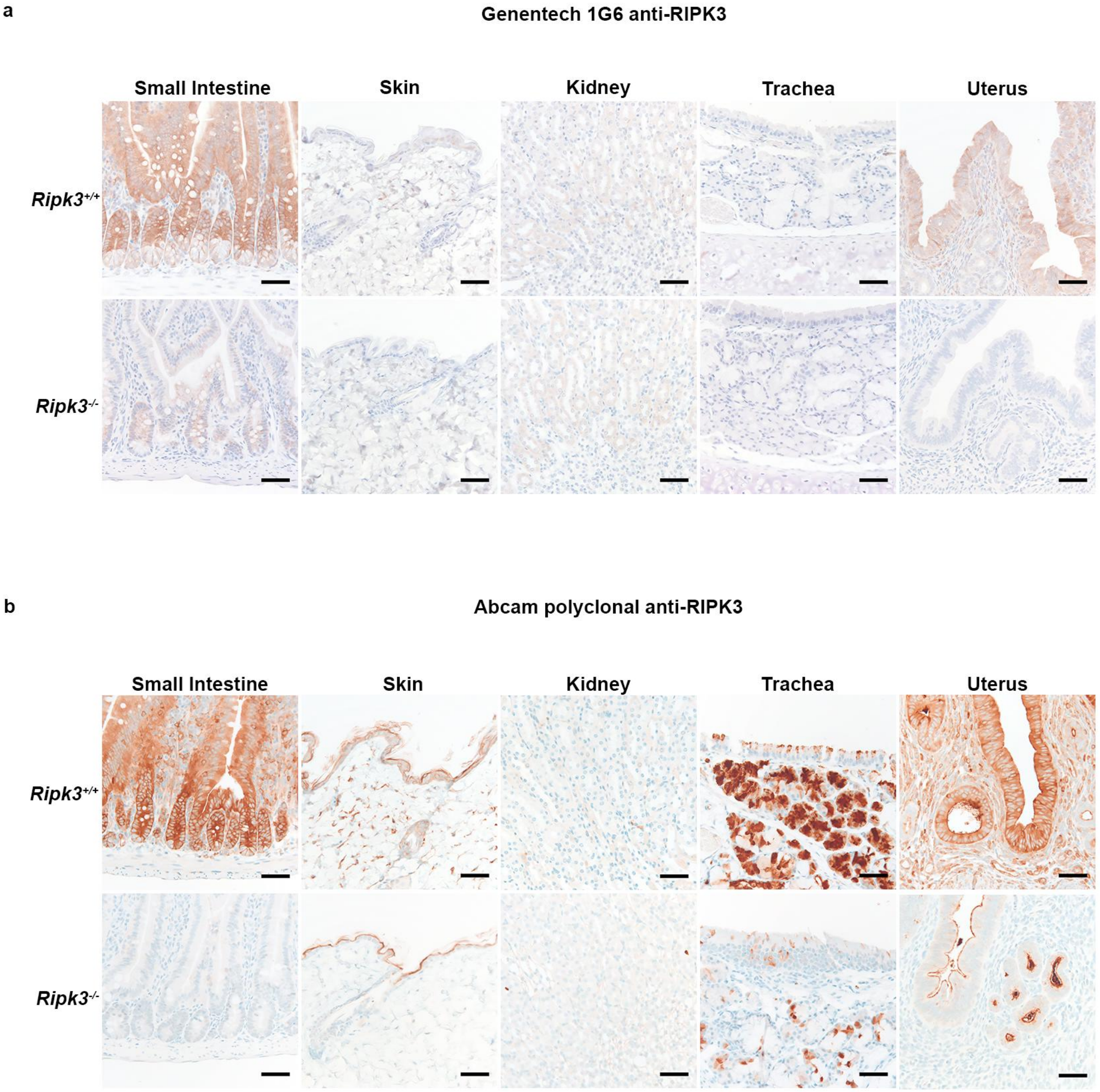

Figure S2. RIPK3 IHC validation with Abcam polyclonal and Genentech 1G6 anti-RIPK3 antibodies.

(a) RIPK3 IHC in the small intestine, skin, kidney, trachea, and uterus of WT and *Ripk3*$^{-/-}$ mice using the Genentech 1G6 anti-RIPK3 antibody. Scale bars, 50 µm. Results are representative of 5 WT and 3 *Ripk3*$^{-/-}$ small intestine, skin, kidneys, and trachea and 2 WT and 1 *Ripk3*$^{-/-}$ uterus.

(b) RIPK3 IHC in the small intestine, skin, kidney, trachea, and uterus of WT and *Ripk3*$^{-/-}$ mice using a polyclonal anti-RIPK3 antibody. Scale bars, 50 µm. Results representative of 5 WT and 3 *Ripk3*$^{-/-}$ small intestines, skin, and kidneys; 3 WT and 3 *Ripk3*$^{-/-}$ trachea; and 2 WT and 1 *Ripk3*$^{-/-}$ uterus.

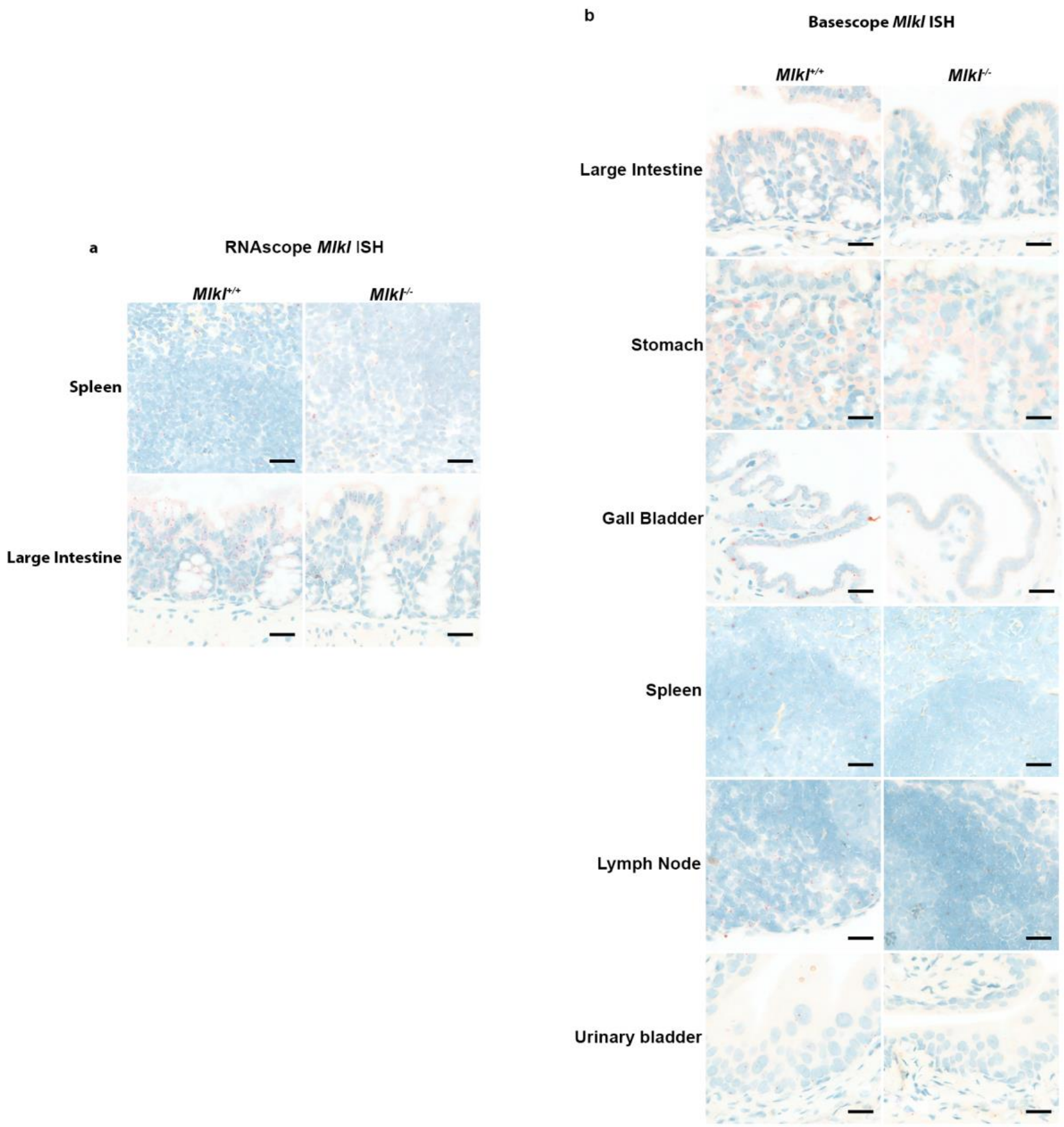

Figure S3. *Mlkl* ISH in WT and *Mlkl*$^{-/-}$ mouse tissues.

(a) *Mlkl* ISH in WT and *Mlkl*$^{-/-}$ spleen and large intestine using RNAscope probes. Partial transcripts are detected in the *Mlkl*$^{-/-}$ tissues. Scale bars, 25 µm. Results representative of 1 mouse per genotype.

(b) *Mlkl* ISH in WT and *Mlkl*$^{-/-}$ mouse tissues using Basescope probes that target exon 3. Scale bars, 25 µm. Results representative of 3 WT and 3 *Mlkl*$^{-/-}$ large intestine, stomach, and lymph node; 2 WT and 2 *Mlkl*$^{-/-}$ gall bladder and spleen; and 2 WT and 1 *Mlkl*$^{-/-}$ urinary bladder.

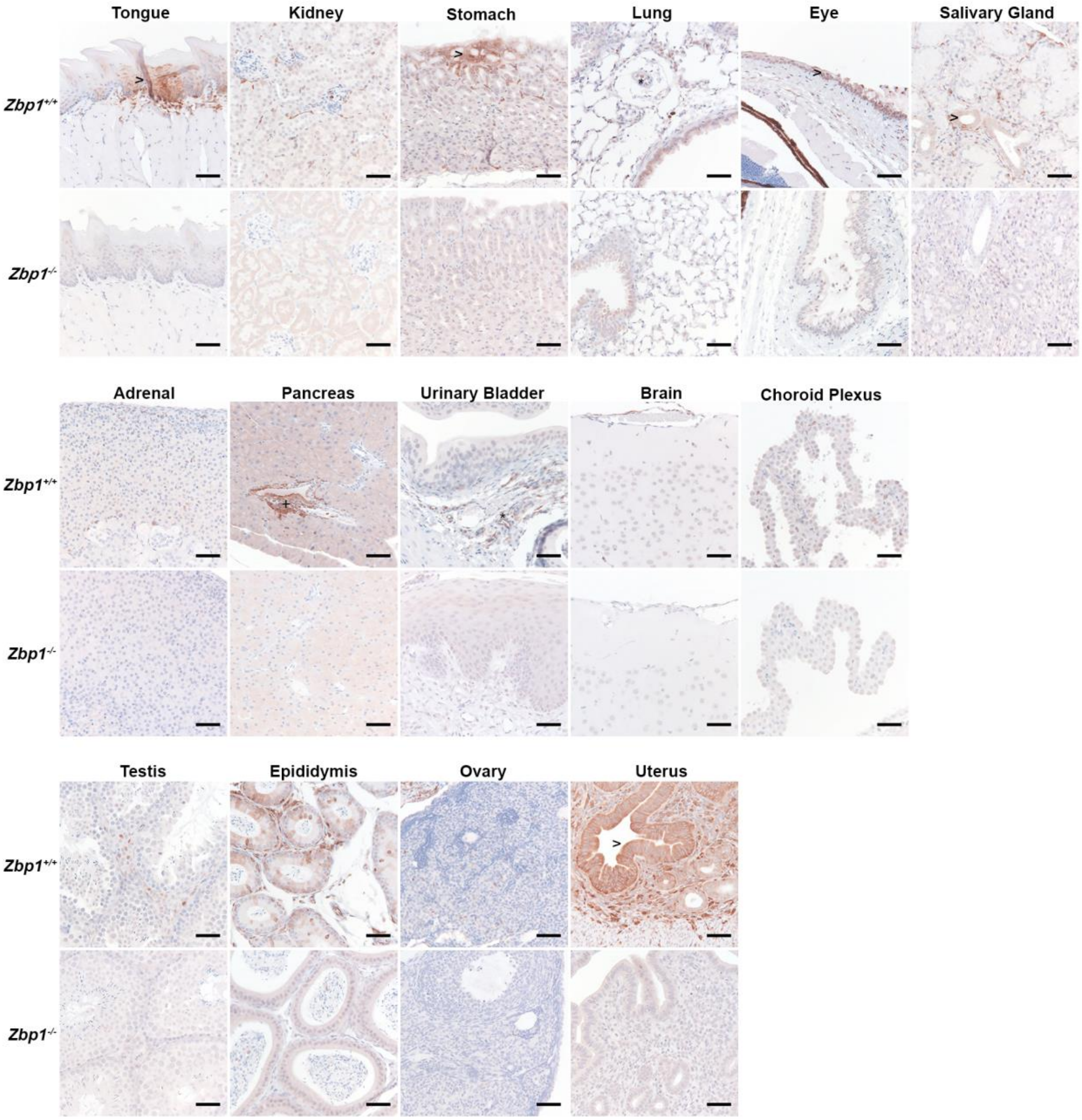

Figure S4. ZBP1 IHC in WT and *Zbp1*$^{-/-}$ mouse tissues. Scale bars, 50 µm. Results representative of 5 WT and 4 *Zbp1*$^{-/-}$ mice for the tongue, kidney, stomach, lung, eye, salivary gland, adrenal, pancreas, urinary bladder, and brain/ choroid plexus; 3 WT and 2 *Zbp1*$^{-/-}$ testis and epididymis; and 2 WT and 2 *Zbp1*$^{-/-}$ ovary and uterus. *, denote endothelial labeling; >, highlights epithelial labeling; and +, highlights immune cell labeling.

a

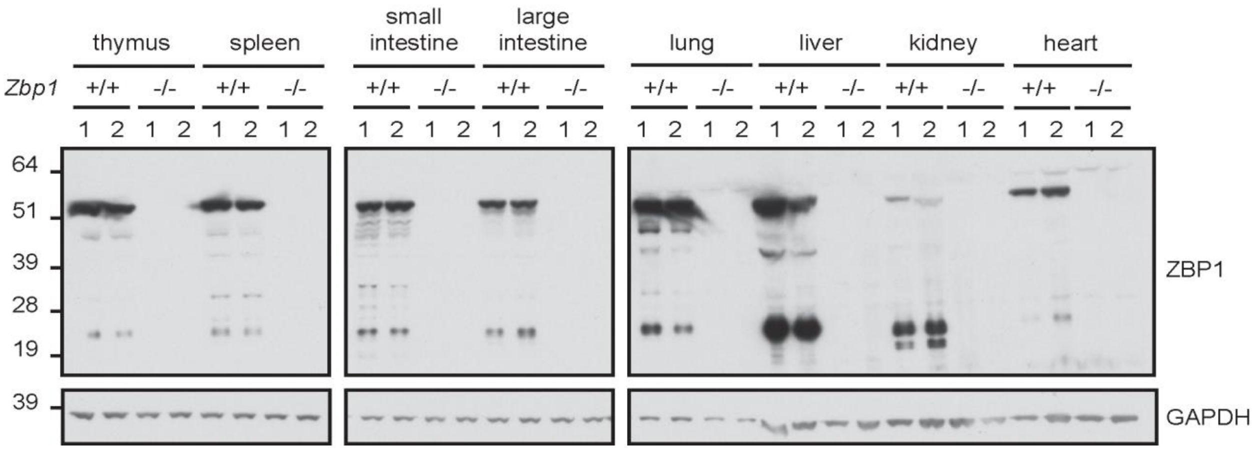

b

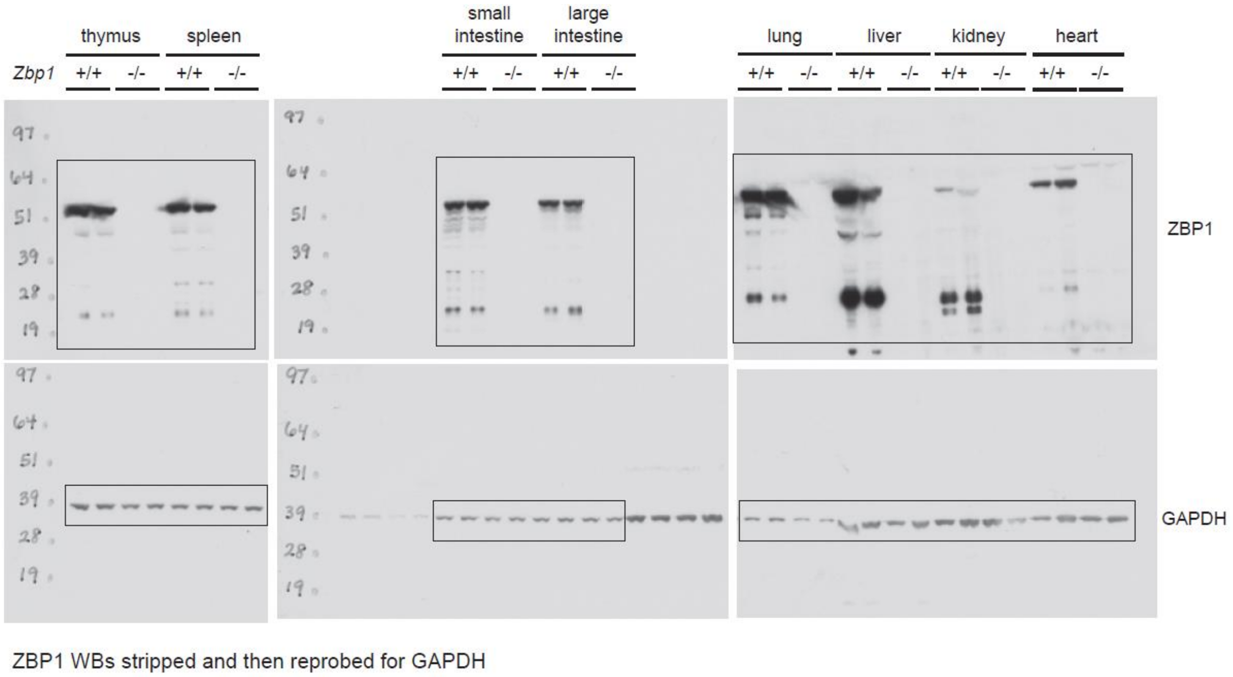

Figure S5. ZBP1 western blots of WT and *Zbp1*$^{-/-}$ mouse tissues. (a) cropped and (b) uncropped images of the blots.

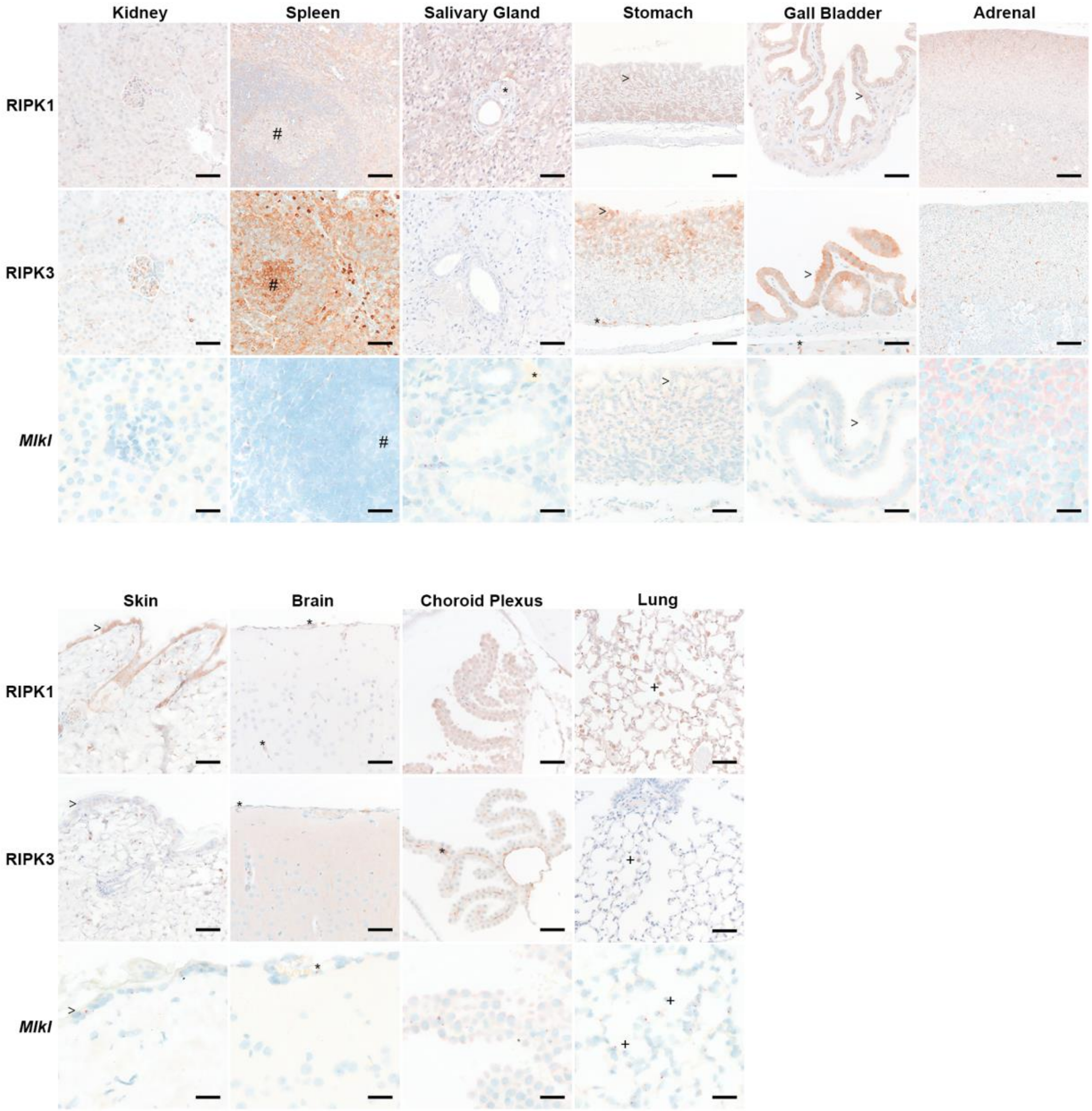

Figure S6. RIPK1 and RIPK3 IHC, and *Mlkl* ISH in WT mouse tissues. Scale bars, 100 µm (RIPK1 and RIPK3: stomach and adrenal), 50 µm (RIPK1 and RIPK3: kidney, spleen, salivary gland, gall bladder, skin, brain, choroid plexus, and lung; *Mlkl*: stomach), or 25 µm (*Mlkl*: kidney, spleen, salivary gland, gall bladder, adrenal, skin, brain, choroid plexus, and lung). Results representative of 5 WT kidney, spleen, salivary gland, stomach, adrenal, skin, and lung and 3 WT gall bladder. *, denote endothelial labeling; #, denote germinal centers; >, highlights epithelial labeling; and +, highlights immune cell labeling.

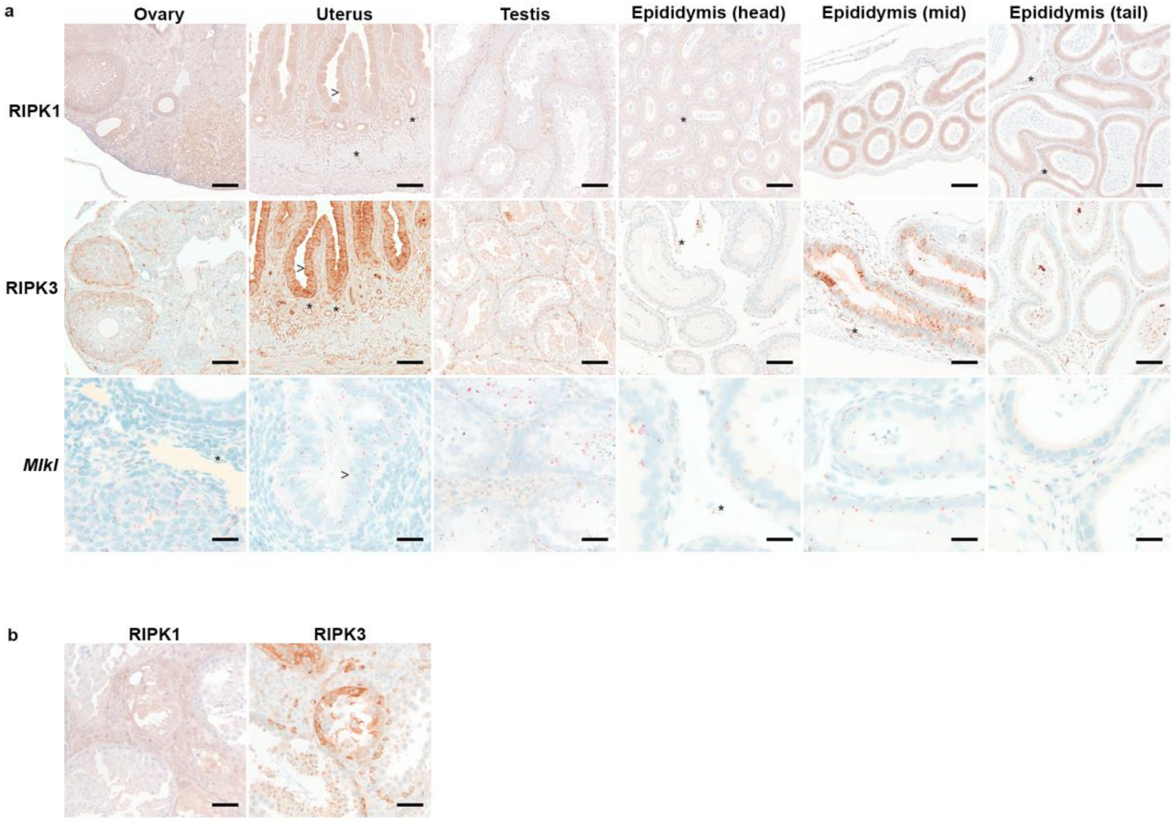

Figure S7. RIPK1 and RIPK3 IHC, and *Mlkl* ISH in WT mouse tissues.

(a) RIPK1 and RIPK3 IHC, and *Mlkl* ISH in WT reproductive organs. Scale bars, 100 µm (RIPK1 and RIPK3) or 25 µm (*Mlkl*). Results are representative of 3 WT testis and epididymis and 2 WT ovary and uterus. *, denote endothelial labeling and >, highlights epithelial labeling.

(b) RIPK1 and RIPK3 IHC in WT testis demonstrating increased labeling associated with degenerate tubules. Scale bars, 50 µm. Results representative of 3 WT testis.

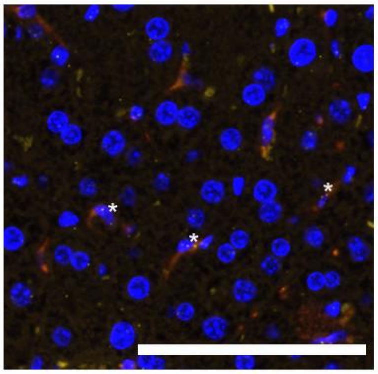

Figure S8. *Mlkl* ISH (yellow spots) and CD68 immunofluorescence (red) in a WT liver (N = 1). *Mlkl* and CD68 labeling co-localizes in Kupffer cells (*). Scale bar, 100 µm. Note there is erythrocyte autofluorescence present in yellow as well.

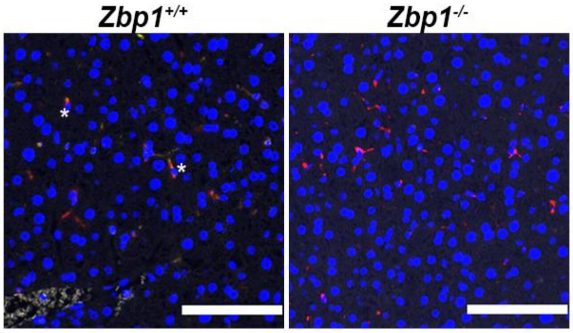

Figure S9. ZBP1 (yellow) and CD68 (red) dual immunofluorescence in WT (N = 1) and *Zbp1*$^{-/-}$ (N = 1) livers. ZBP1 and CD68 co-localize in Kupffer cells of the WT liver, but no ZBP1 labeling is present in the *Zbp1*$^{-/-}$ liver. Scale bars, 100 µm.

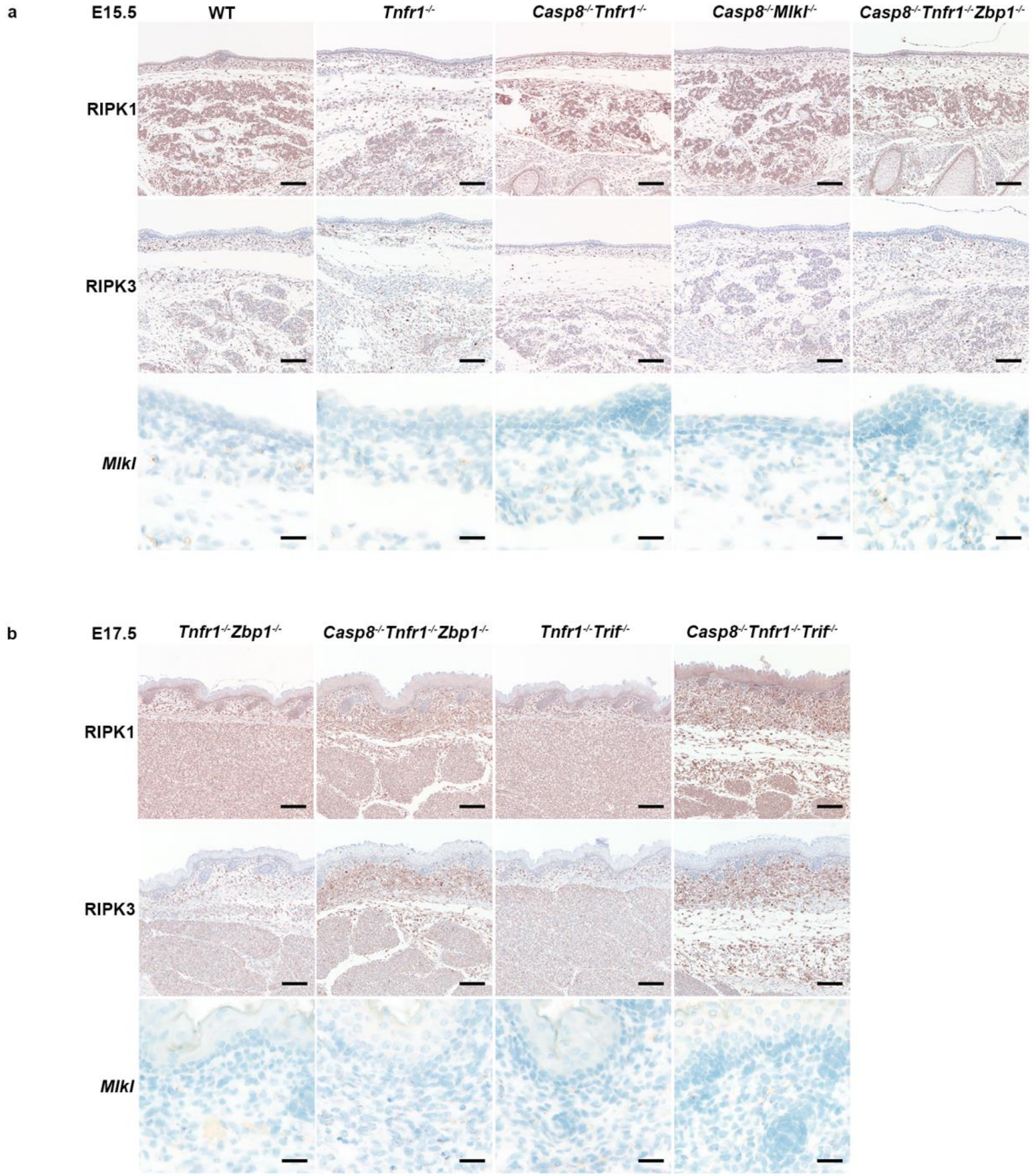

Figure S10. RIPK1 and RIPK3 IHC, and *Mlkl* ISH in mouse embryonic skin.

(a) E15.5 embryos. RIPK1, RIPK3, and *Mlkl* labeling is not strikingly different between genotypes at this age. Scale bars, 100 µm (RIPK1 and RIPK3) or 25 µm (*Mlkl*). Results representative of 3 embryos per genotype.

(b) E17.5 embryos. Dermal inflammation in *Casp8*$^{-/-}$*Tnfr1*$^{-/-}$*Zbp1*$^{-/-}$ and *Casp8*$^{-/-}$*Tnfr1*$^{-/-}$*Trif*$^{-/-}$ embryos is associated with expansion of the dermis by RIPK1, RIPK3, and *Mlkl* expressing cells. Increased RIPK1 labeling is also apparent in the hyperplastic

epidermis. Scale bars, 100 µm (RIPK1 and RIPK3) or 25 µm (*Mlkl*). Results representative of 3 *Tnfr1*$^{-/-}$*Zbp1*$^{-/-}$, *Casp8*$^{-/-}$*Tnfr1*$^{-/-}$*Zbp1*$^{-/-}$, and *Casp*$^{-/-}$*Tnfr1*$^{-/-}$*Trif*$^{-/-}$ embryos and 2 *Tnfr1*$^{-/-}$*Trif*$^{-/-}$ embryos.
