# Supplementary material for: ZBP1 and TRIF trigger lethal necroptosis in mice lacking caspase-8 and TNFR1
Source: Cell Death Differ. 2024 Mar 28;31(5):672–82. doi: 10.1038/s41418-024-01286-6 (PMC11093969; doi:10.1038/s41418-024-01286-6)
Supplement: Supplementary file 2 — Supplementary Methods [file 41418_2024_1286_MOESM2_ESM.pdf]

Supplementary Materials and Methods

ZBP1 and TRIF trigger lethal necroptosis in mice lacking caspase-8 and TNFR1

Solon et al.

IHC

For RIPK1 IHC, sections were placed in Target retrieval solution, citrate pH 6 (Agilent, Santa Clara, CA, USA) for 20 min at 99°C, and then 3% hydrogen peroxide in PBS for 4 min at room temperature (RT). Labeling was with 3.5 µg/ml 10C7 rat anti-mouse RIPK1 (Genentech, South San Francisco, CA, USA) in Tris-NaCl blocking buffer (Perkin Elmer, Shelton, CT, USA) for 1 h at RT, followed by rabbit anti-rat IgG (H+L) (Vector Labs, Newark, CA, USA) for 30 min, and PowerVision Poly-HRP anti-rabbit (Leica Biosystems, Deer Park, IL, USA) for 30 min. Sections were developed in metal-enhanced diaminobenzidine (DAB; Thermo Scientific, Rockford, IL, USA) and counterstained with hematoxylin.

For ZBP1 IHC, sections were placed in EDTA pH 8 retrieval solution (Abcam, Waltham, MA, USA; ab93680) for 20 min at 99°C, and 3% hydrogen peroxide in PBS for 4 min at RT. Endogenous biotin was blocked using the Biotin blocking kit following manufacturer's recommendations (ScyTek Laboratories, Logan, UT) followed by PBS containing 10% normal donkey serum and 3% BSA. Labeling was with 7.5 µg/ml GN58.3 rat anti-mouse ZBP1 (Genentech) in PBS containing 10% normal donkey serum and 3% BSA for 1 h at RT, and then biotinylated donkey anti-rat (Jackson ImmunoResearch, West Grove, PA, USA) for 30 min, followed by ABC-HRP (Vector Labs) for 30 min with DAB and hematoxylin counterstain.

Labeling with the rabbit anti-RIPK3 polyclonal (Abcam) was performed on the Ventana Discovery Ultra autostainer. Sections were treated with Cell Conditioning 1 solution for 64 min at 97°C and labeled with 2 µg/ml polyclonal antibody in Ventana antibody diluent with casein (Roche Tissue Diagnostics) for 1 h at 37°C, followed by Ventana Omnimap anti-rabbit HRP for 16 min. Ventana DAB and Hematoxylin II were used for detection and counterstain. Alternatively, sections were treated with Target retrieval solution, citrate pH 6 for 20 min at 99°C, and then 3% hydrogen peroxide in PBS. Endogenous biotin was blocked using the Biotin blocking kit (ScyTek Laboratories) and then PBS containing 3% BSA and 10% rabbit serum. Labeling was with 7.5 µg/ml 1G6 rat anti-RIPK3 (Genentech) in PBS containing 3% BSA and 10% rabbit serum for 1 h at RT, followed by 30 min with biotinylated rabbit anti-rat antibody (Jackson ImmunoResearch), and 30 min in ABC-HRP with DAB and hematoxylin counterstain.

ISH

20zz probes targeting *Mlkl* base pairs 737-1699 or *Ripk1* base pairs 267-1218 (Advanced Cell Diagnostics [ACD], Newark, CA, USA) were applied on the BondRx platform (Leica Biosystems) autostainer. Standard LSx RNAScope Red detection procedures using RNAscope 2.5 LSx Reagent Kit-RED included a 45 min AMP5 time, and pretreatment with ER2 at 95°C for 15 min followed by ACD enzyme for 15 min. Basescope ISH for *Mlkl* exon 3 used a 1zz probe targeting base pairs 816-864 (ACD). Standard LS Basescope Red detection procedures using BaseScope LS Reagent and BOND Polymer Refine Red Detection plus Hematoxylin included an AMP7 time

of 30 min, and pretreatment with ER2 at 95°C for 15 min, followed by ACD enzyme for 15 min. Controls included *Ppib* and *Dapb* probes.

CD68 and ZBP1 dual immunofluorescence

Immunofluorescence for CD68 and ZBP1 was performed on 4 µm thick FFPE sections of mouse liver on the Ventana Discovery Ultra (Roche Tissue Diagnostics, Tucson, AZ, USA) autostainer. Sections were pretreated with Cell Conditioning 1 (Roche) for 64 minutes at 97C followed by peroxidase blocking using Discovery Inhibitor (Roche) for 8 min. Sections were incubated with anti-CD68 rabbit polyclonal antibody (Abcam, Waltham, MA) at 0.5 µg/ml in 3% BSA/PBS for 32 min at RT. Sections then were incubated with Ventana Omnimap Anti-Rabbit HRP for 16 min followed by Discovery Cy5 for 12 min. Elution was performed using CC2 retrieval for 8 min at 100°C. Sections were then incubated with anti-ZBP1 rat monoclonal antibody (Clone 58.3; Genentech, South San Francisco, CA, USA) at 7.5 µg/ml in 3% BSA/PBS for 60 min at RT. Sections were incubated in rabbit anti-rat (Vector Labs, Newark, CA) at 5 µg/ml in 3% BSA/PBS for 32 minutes followed by Ventana Omnimap Anti-Rabbit HRP for 16 min. Sections were then incubated with Discovery Rhodamine 6G for 12 min followed by counterstain with DAPI (Invitrogen, Waltham, MA) and coverslipped. Isotype control antibodies were used to confirm specificity.

Mlkl fluorescent ISH and CD68 immunofluorescence

All ISH and immunofluorescence steps were carried out on the BondRx platform (Leica Biosystems, Deer Park, IL, USA) autostainer. In situ hybridization for *Mlkl* was performed on 4 µm thick FFPE mouse liver using a 20zz probe targeting base pairs 737-1699 (Advanced Cell Diagnostics, Newark, CA, USA). ISH incubation times and steps were performed following the standard RNAScope 2.5 LS Assay- Red detection system procedures up to AMP4. After AMP4, sections were incubated with Opal 570 (Akoya, Menlo Park, CA) at 1:1000 for 30 min. Sections were incubated with 3% hydrogen peroxide in PBS followed by anti-CD68 rabbit polyclonal antibody (Abcam, Waltham, MA) at 0.5 µg/mL in 3% BSA/PBS for 60 min at RT. Sections were then incubated in goat anti-rabbit HRP (Revvity, Waltham, MA) at 1:1000 in 3% BSA/PBS for 30 min followed by Opal 690 at 1:1000 for 30 min. Sections were counterstained with DAPI (Invitrogen, Waltham, MA) and coverslipped.
